# B cells sustain tumor-specific CD4 T cells to promote response to PD-1 targeted therapy

**DOI:** 10.64898/2026.09.18.751292

**Published:** 2026-09-24

**Authors:** Abishek Vaidya, Etienne Humblin, Nataliya Prokhnevska, Verena van der Heide, Laurine Binet, Jachym Harwood, Raphaël Mattiuz, Anthony Lozano, Pauline Hamon, Bruno Cogliati, Simon Goldstein, Isabel Korpas, Katherine E. Lindblad, Suhaana Sriram, Harald Hartweger, Glaucia Furtado, Sacha Gnjatic, Edgar Gonzalez-Kozlova, Brad R. Rosenberg, Amaia Lujambio, Miriam Merad, Michel C. Nussenzweig, Alice O. Kamphorst

## Abstract

Immunotherapy transformed cancer treatment, yet precise correlates of response remain to be defined. While intratumoral follicular helper like (Tfhl) CD4 T cells and IgG1 ^+^ B cells have been associated with improved outcomes to PD-1 blockade for hepatocellular carcinoma (HCC), their mechanisms contributing to response are unclear. To address this question, we developed a murine model of HCC and examined CD8, CD4 and B cell responses. Increasing CD4-help sensitizes mice to PD-1 blockade, in a CD4- and CD8-dependent manner. Tumor-specific CD4 T cells contained Tfhl and Th1 populations, and both *Bcl6* and T-bet were required for efficacy. Antigen-specific B cells were essential for CD4-helper expansion, yet secretion of antibodies was not required for long-term survival. Thus, B cells are critical for effective immunotherapy, but secreted antibodies are not.

## Full Text Availability

The license terms selected by the author(s) for this preprint version do not permit archiving in PMC. The full text is available from the preprint server.

